# Atorvastatin exhibits anticancer effects by inhibiting YAP/TAZ activity in mesenchymal-like non-small cell lung cancer

**DOI:** 10.1038/s41598-025-15624-2

**Published:** 2025-08-18

**Authors:** Takuro Ishikawa, Tomohito Okubo, Natsuki Matsushita, Jiro Tashiro, Katsuhiko Warita, Munekazu Naito

**Affiliations:** 1 Department of Veterinary Anatomy, School of Veterinary Medicine, Tottori University, 4-101 Koyama Minami, Tottori, Tottori 680-8553 Japan; 2 Department of Anatomy, School of Medicine, Aichi Medical University, 1-1 Yazakokarimata, Nagakute, Aichi 480-1195 Japan; 3 Division of Laboratory Animal Research, School of Medicine, Aichi Medical University, 1-1 Yazakokarimata, Nagakute, Aichi 480-1195 Japan

**Keywords:** Statins, Non-small cell lung cancer, YAP, TAZ, Mesenchymal cancer cells, Cancer, Cell biology

## Abstract

Non-small cell lung cancer (NSCLC) accounts for most lung cancer diagnoses. Statins preferentially inhibit the proliferation of mesenchymal- over epithelial-like cells in various types of cancer, including NSCLCs. However, the mechanisms underlying the differential statin sensitivity of mesenchymal and epithelial cancer cells remain unknown. Statins inhibit YAP/TAZ, effectors of the Hippo pathway, via depletion of geranylgeranyl pyrophosphate. Here, we aimed to elucidate the mechanisms underlying statin sensitivity in mesenchymal cancer. We explored the anticancer effects of atorvastatin and its association with YAP/TAZ activity in NSCLC cell lines with different epithelial-mesenchymal phenotypes. Atorvastatin significantly reduced the proliferation, migration, and invasion of mesenchymal-like cells, while showing negligible effect on epithelial-like cells. Atorvastatin also inhibited YAP/TAZ nuclear localization and downstream gene expression in mesenchymal cells but did not affect epithelial cells. Small interfering (si) RNA-mediated inhibition of both YAP and TAZ reduced the proliferation of all NSCLC cell lines tested, regardless of phenotype, indicating that sensitivity to YAP/TAZ inhibition and statins differ. In summary, our results suggest that inhibited YAP/TAZ nuclear localization by statins differs between epithelial and mesenchymal NSCLC cell lines, resulting in differential statin sensitivity.

## Introduction

Lung cancer is one of the most prevalent cancers worldwide. In 2022, an estimated 2.5 million new cases of lung cancer were diagnosed, and 1.8 million people died from the disease in 185 countries, making it the leading cause of cancer-related deaths^1^. Lung cancer is classified into small cell lung cancer and non-small cell lung cancer (NSCLC) based on its biology, therapy, and prognosis, with NSCLC accounting for more than 85% of all lung cancer cases^2^. Approximately 30–35% of patients with lung cancer have metastatic disease at the time of diagnosis^3^. Treatment for metastatic NSCLC primarily involves chemotherapy and palliative radiotherapy, and response to chemotherapy is considered a critical prognostic factor^4^. However, NSCLC is often resistant to chemotherapy, which renders its treatment challenging^5^.

Statins are widely used for the treatment of dyslipidemia. Research on the anticancer effects of statins has progressed in recent years, and many epidemiological studies have reported the association of statins with reduced cancer-related mortality^6,7^. In addition, clinical intervention studies have shown that statins can suppress tumor growth and promote tumor apoptosis^8,9^. Statins inhibit 3-hydroxy-3-methylglutaryl-CoA reductase, the rate-limiting enzyme in the mevalonate pathway, thereby reducing the synthesis of cholesterol and multiple intermediate metabolites. The inhibition of geranylgeranyl pyrophosphate (GGPP), an intermediate, has been reported to play a key role in the anticancer effects of statins^10^. GGPP is required for post-translational modification of proteins by prenylation, and small GTPases, which are involved in intracellular processes such as proliferation, apoptosis, and migration, are prenylated. Therefore, statins are believed to exert their anticancer effects by inhibiting prenylation of small GTPases^11^.

Yes-associated protein 1 (YAP1) and transcriptional coactivator with PDZ-binding motif (TAZ, also known as WWTR1) are transcriptional coactivators and effectors of the Hippo signaling pathway. The Hippo pathway regulates organ size and wound healing by modulating cell proliferation and apoptosis. The Hippo pathway is activated under low mechanical stress or high cell density conditions, leading to the phosphorylation of YAP/TAZ by LATS1/2 kinases, which causes the translocation of YAP/TAZ from the nucleus to the cytoplasm^12,13^. Conversely, YAP/TAZ are dephosphorylated and accumulate in the nucleus when the Hippo pathway is inactivated, where they interact with the TEAD family transcription factors to promote the transcription of target genes^14,15^. Activation of YAP/TAZ is frequently observed in various types of cancers, contributing to tumor progression and poor prognosis^16^. YAP/TAZ promote the transcription of genes involved in cell survival, proliferation, motility, and resistance to apoptosis. Rho small GTPase, prenylated by GGPP, activates YAP/TAZ by inhibiting their phosphorylation and promoting their nuclear accumulation^17^. Statins exert anticancer effects by inhibiting YAP/TAZ activity in several cancer cells, including hepatocellular carcinoma, mesothelioma, and pancreatic cancer^18–20^.

Previously, we demonstrated that statins preferentially attenuate the proliferation of mesenchymal-like cancer cells over epithelial-like cells across various cancer types, including lung cancer^21,22^. In general, cancer cells undergo epithelial-mesenchymal transition (EMT), a process via which epithelial cancer cells transform into a mesenchymal phenotype and acquire malignant properties, such as metastatic potential and drug resistance^23^. Therefore, statins have the potential to suppress invasion and metastasis of highly malignant cancers that have undergone EMT. However, the mechanisms underlying the sensitivity of mesenchymal cancer cells to statins remain unclear.

Here, we investigated the effects of atorvastatin on NSCLC cell lines with different epithelial-mesenchymal phenotypes. We elucidated part of the mechanisms responsible for the differences in statin sensitivity between epithelial and mesenchymal cancer cells. Our results showed that NSCLC cell lines are susceptible to the concomitant inhibition of YAP and TAZ regardless of their epithelial-mesenchymal phenotype. We also found that the amount of inhibited YAP/TAZ nuclear localization by atorvastatin differs between epithelial and mesenchymal NSCLC cell lines, which leads to different sensitivity to statin. Our findings should help to understand the anticancer mechanisms of statins and lead to their effective use.

## Results

### Assessment of atorvastatin sensitivity and epithelial-mesenchymal phenotype in NSCLC cell lines

**Fig. 1:**
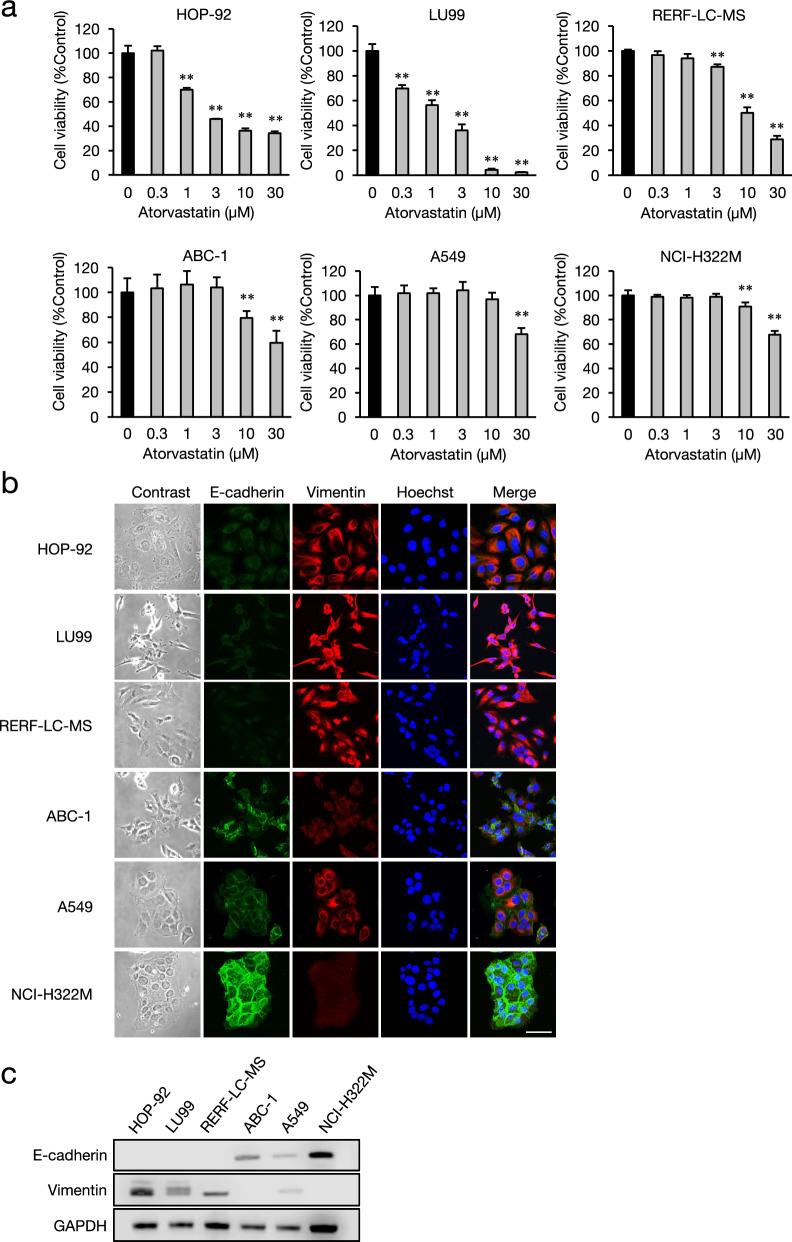
Sensitivity to atorvastatin and expression status of E-cadherin and vimentin in NSCLC cell lines. (**a**) NSCLC cell lines were treated with 0.3–30 μM atorvastatin or DMSO vehicle control and cell viability was determined at 48 h using a CCK-8 assay. Each value represents the mean ± SD (n = 3). Data were analyzed using the Dunnett’s test: **p < 0.01 compared to the vehicle control group. (**b**) Images of cells immunostained for E-cadherin (green) and vimentin (red), as well as with Hoechst 33342 (blue). Scale bar: 50 μm. (**c**) E-cadherin and vimentin levels in cell lines were determined using western blotting. GAPDH was used as the loading control. Cropped images of the western blot are shown and original uncropped blots are presented in Supplementary Fig. S1.

Statin-sensitive cancer cells have mesenchymal-like characteristics, such as abundant cytoplasmic expression of vimentin and no E-cadherin on the plasma membrane^21^. We evaluated statin sensitivity as well as vimentin and E-cadherin expression in six NSCLC cell lines. Atorvastatin dose-dependently decreased the viability of the NSCLC cell lines to different degrees. Atorvastatin at concentrations of 1 and 0.3 μM significantly reduced the viability of the HOP-92 and LU99 cells, respectively (Fig. 1a). Atorvastatin (3 μM) reduced the viability of RERF-LC-MS cells, whereas 10, and 30 μM significantly reduced that of ABC-1, A549, and NCI-H322M. We used immunofluorescence microscopy and western blotting to identify the expression and localization of epithelial-mesenchymal markers and the epithelial-mesenchymal phenotypes of these cell lines (Fig. 1b, c). The expression of vimentin (mesenchymal marker) and E-cadherin (epithelial marker) was respectively high and undetectable in HOP-92, LU99, and RERF-LC-MS cells. Vimentin and membrane E-cadherin were both expressed in A549 cells, whereas ABC-1 and NCI-H322M cells expressed membrane E-cadherin but little vimentin. Consistent with the epithelial-mesenchymal marker expression, HOP-92, LU99, and RERF-LC-MS cells with low E-cadherin expression had loose cell–cell adhesions and small contact areas, whereas the other three cell lines that expressed membrane E-cadherin (especially NCI-H322M), tended to have tight intercellular adhesions. Taken together, these results indicated that mesenchymal-like NSCLC cells tend to be sensitive to statin, whereas epithelial-like NSCLC cells, with membrane expression of E-cadherin, tend to be relatively statin-resistant. This trend was similar in prostate cancer (PC) cells (Supplementary Fig. S2, S3). Mesenchymal-like PC-3 cells were more sensitive to statins than DU-145 cells expressing membrane E-cadherin.

### Effects of atorvastatin on cell migration and invasion

**Fig. 2:**
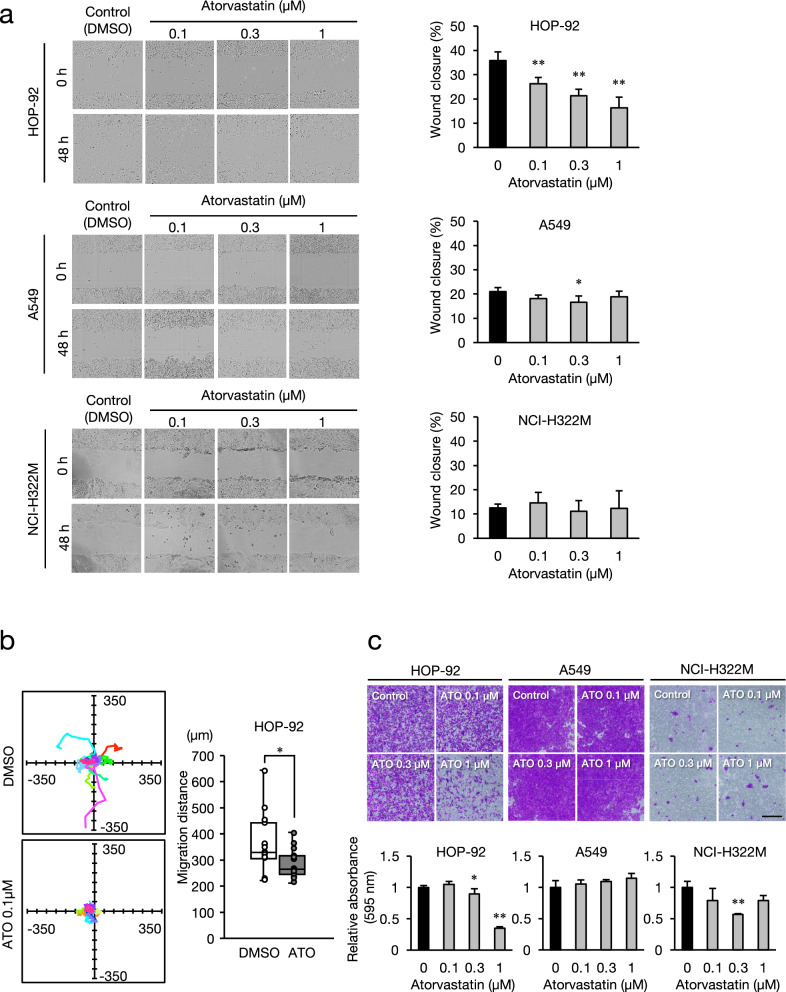
Effect of atorvastatin on migration and invasion of NSCLC cell lines. (**a**) Effects on cell migration were measured in a wound healing assay. Confluent cells were scratched and cultured with 0.1–1 µM atorvastatin or DMSO for 48 h. Wound closure (%) was calculated based on the images at 0 and 48 h. Each value represents the mean ± SD (n = 3). (**b**) Effect on single-cell motility of HOP-92 cells was analyzed using the IncuCyte ZOOM imaging system. Low-density cells were treated with 0.1 µM atorvastatin (ATO) or DMSO and individual cells (n = 15) were tracked for 48 h using time-lapse imaging. Box plots show the migrated distance (µm). (**c**) The effect on cell invasion in NSCLC cell lines was measured using the Matrigel-coated transwell assay. Cells were incubated in transwell inserts with 0.1–1 µM ATO or DMSO. The cells that migrated into the lower chamber were stained with crystal violet at 48 h after plating and quantified by measuring the absorbance. Scale bar: 1.0 mm. Each value represents the mean ± SD (n = 3). Data were analyzed using the Dunnett’s test (**a**, **c**) and two-tailed Welch’s t-test (**b**): *p < 0.05 and **p < 0.01 compared to the vehicle control group.

We investigated the effects of atorvastatin on NSCLC cell migration using wound-healing assays (Fig. 2a). Atorvastatin dose-dependently reduced the migration of HOP-92, but not that of A549 or NCI-H322M cells. We evaluated the effects of atorvastatin on the motility of single HOP-92 cells using cell-tracking (Fig. 2b). The results showed that atorvastatin (0.1 µM) significantly reduced the motility of single HOP-92 cells. We evaluated the invasive ability of each cell line using transwell assays. Atorvastatin inhibited HOP-92 cell invasion into Matrigel to 35% of that of the control (Fig. 2c), but did not inhibit A549 and NCI-H322M cell invasiveness. A few NCI-H322M cells incubated without (control) and with statin were invasive.

## Effects of atorvastatin on YAP/TAZ activity

**Fig. 3:**
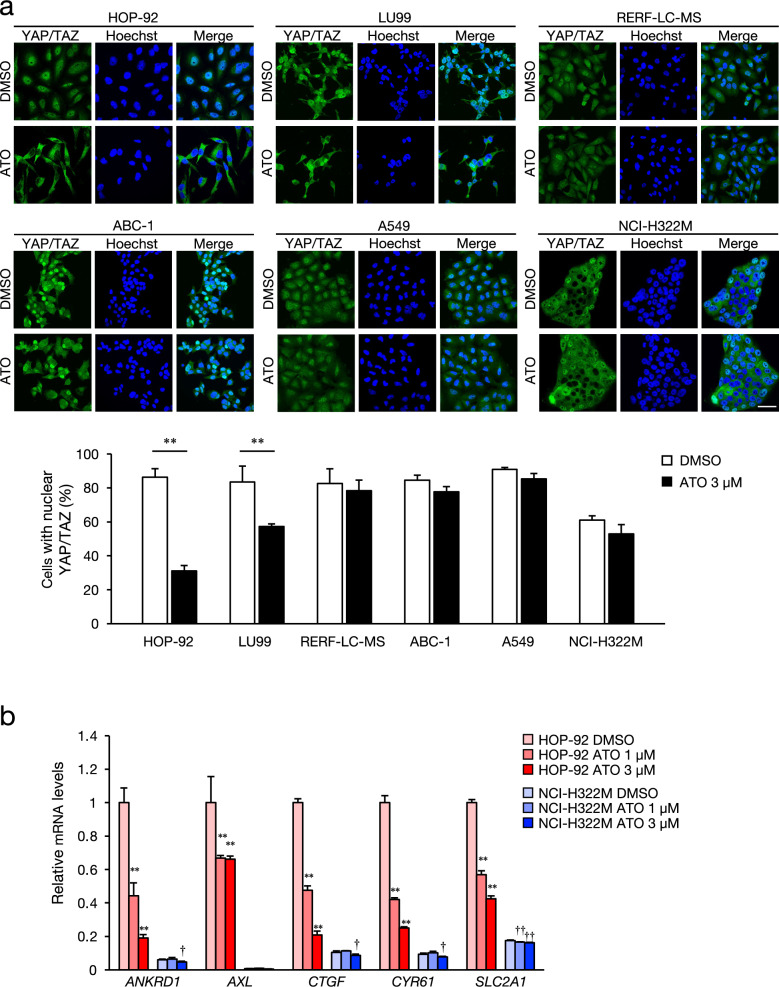
Effect of atorvastatin on YAP/TAZ activity in NSCLC cell lines. (**a**) Representative images of cells immunostained for YAP/TAZ (green) and Hoechst 33342 (blue) in NSCLC cell lines treated with 3 µM atorvastatin (ATO) or DMSO for 24 h. Bars show quantification of cells with nuclear YAP/TAZ. Each value represents the mean ± SD (n = 3; Over 250 cells were scored). Data were analyzed using two-tailed Welch’s t-test: **p < 0.01 compared to the vehicle control group. Scale bar: 50 μm. (**b**) Effect of atorvastatin on YAP/TAZ target gene expression in HOP-92 and NCI-H322M cells treated with 1 µM and 3 µM ATO or DMSO for 24 h. Data are shown as relative values, with the expression level of HOP-92 cells treated with DMSO set at 1. Each column represents the mean ± SD (n = 3). Data were analyzed using the Dunnett’s test: **p < 0.01 compared to the vehicle control group of HOP-92, and †*p* < 0.05 and ††*p* < 0.01 compared to the vehicle control group of NCI-H322M.

**Fig. 4:**
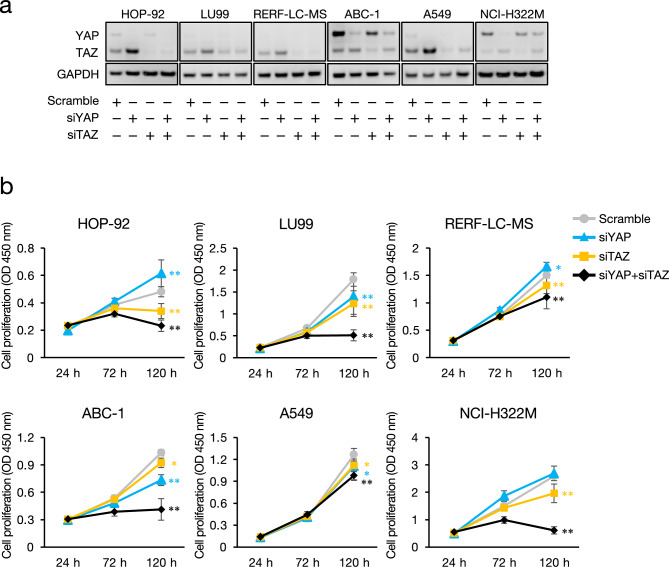
Effect of YAP/TAZ knockdown in NSCLC cell lines. (**a**) YAP and TAZ protein expression in NSCLC cells treated with siRNA targeting YAP and/or TAZ or scrambled control for 72 h. GAPDH was used as the loading control. Cropped images of the western blot are shown and original uncropped blots are presented in Supplementary Fig. S6. (**b**) Growth curve of NSCLC cells treated with siRNA targeting YAP and/or TAZ or scrambled control. Each value represents the mean ± SD (n = 3). Data were analyzed using the Dunnett’s test: *p < 0.05 and **p < 0.01 compared to the scrambled control group.

Atorvastatin suppressed the nuclear localization of YAP/TAZ in statin-sensitive HOP-92 and LU99 cells, but had little effect on RERF-LC–MS, ABC-1, A549, and NCI-H322M cells that are relatively low sensitivity to statin (Fig. 3a). The findings were similar in PC cells (Supplementary Fig. S4). Atorvastatin inhibited the nuclear localization of YAP/TAZ in statin-sensitive PC-3 cells but not in statin-resistant DU-145 cells. Atorvastatin dose-dependently decreased mRNA expression of the YAP/TAZ target genes, *ANKRD1*, *AXL*, *CTGF* (*CCN2*), *CYR61* (*CCN1*), and *SLC2A1* in HOP-92 cells but were somewhat altered in NCI-H322M cells (Fig. 3b). Moreover, the basal expression of the YAP/TAZ target genes was higher in HOP-92 cells, than in NCI-H322M cells (Fig. 3b). This trend was supported by the results of analyzing RNA expression data in 143 NSCLC cell lines downloaded from the Cancer Cell Line Encyclopedia (CCLE) database^24^. More YAP/TAZ target genes^25^ were expressed by mesenchymal- than epithelial-like cells (Supplementary Fig. S5). The simultaneous knockdown of YAP and TAZ by siRNA reduced the proliferation of all analyzed NSCLC cell lines to varying degrees (Fig. 4a, b). When either YAP or TAZ was knocked down, TAZ inhibition reduced the proliferation of all tested cell lines, whereas that of YAP resulted in reduced proliferation of the LU99, ABC-1, and A549 cells. In addition, siYAP increased TAZ protein levels in all cells except ABC-1, suggesting a compensatory mechanism that involves YAP and TAZ (Fig. 4a). Knockdown of YAP and TAZ using different siRNAs confirmed the absence of off-target effects (Supplementary Fig. S7).

### Anticancer effect of atorvastatin on mesenchymal-like NSCLC cells in vivo

**Fig. 5:**
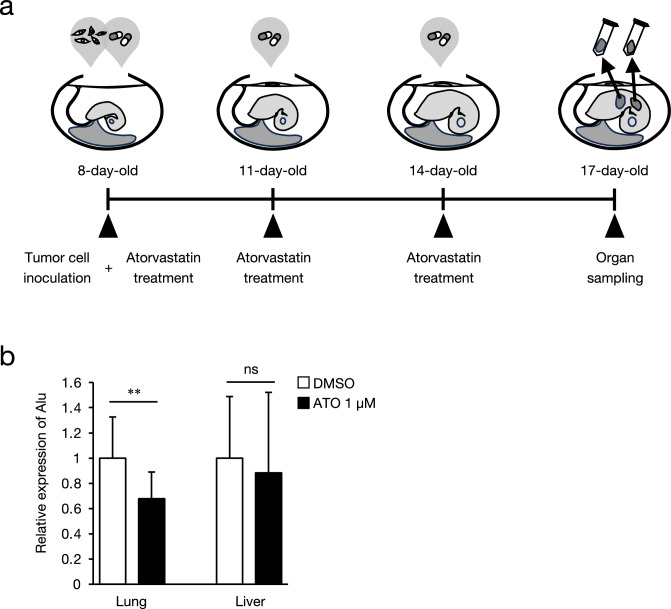
Effect of atorvastatin on metastasis of HOP-92 cells in chicken embryo chorioallantoic membrane assay. (**a**) Schematic diagram of the chicken embryo chorioallantoic membrane (CAM) assay for quantitative detection of metastasis. HOP-92 cells (1 × 10^6^ cells/egg) were inoculated into CAM of 8-day-old embryonated chicken eggs and treated with 1 µM atorvastatin or DMSO. Atorvastatin or DMSO was administered again at 11 and 14 days of age, and organs were harvested from 17-day-old eggs. (**b**) Real-time PCR against human Alu repeats in lungs and livers of chick embryos was performed to analyze metastasis of HOP-92 cells. Data were normalized to chicken *GAPDH* mRNA expression and are shown as relative values, with the expression level of samples treated with DMSO set at 1. Each value represents the mean ± SD (n = 14) and was analyzed using two-tailed Welch’s t-test: ** p < 0.01 compared to the vehicle control group. ns: no significance.

**Fig. 6:**
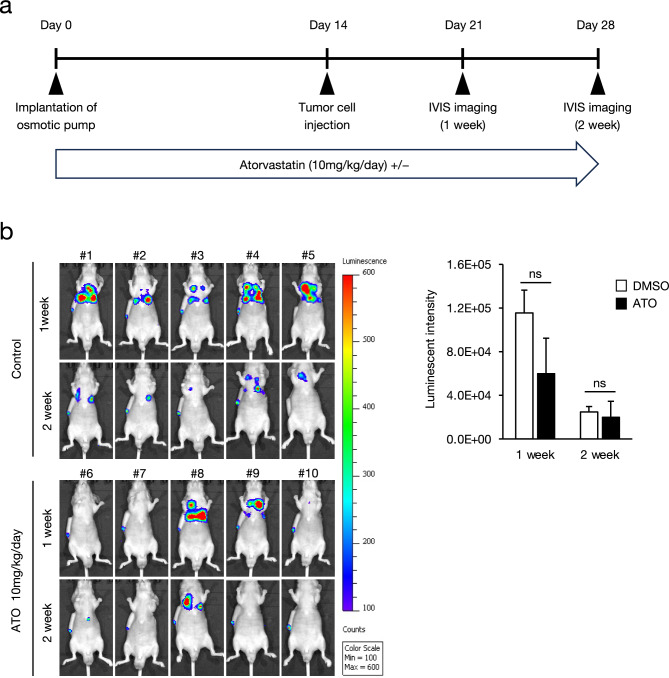
Effect of atorvastatin on tumor survival of HOP-92 cells in mouse model. (**a**) Schematic diagram of the tumor growth experiment in mouse model. Eight-week-old male BALB/c mice (n = 5) were implanted with an osmotic pump to continuously administer atorvastatin (10 mg/kg/day) or DMSO. HOP-92-Luc cells (1 × 10^6^ cells/mouse) were inoculated into the pleural cavity on day 14, and images were captured using an in vivo 3D imaging system (IVIS) after 1 and 2 weeks. (**b**) Images of mice inoculated with HOP-92-Luc cells captured using IVIS. The bars indicate the intensity of luminescence from HOP-92-Luc cells in mice. Each value represents the mean ± SD (n = 5) and was analyzed using two-tailed Welch’s t-test. ns: no significance.

The chorioallantoic membrane (CAM) assay mimics the invasion and metastasis of cancer cells in the body^26^. In the CAM assay performed according to the schedule shown in Fig. 5a, atorvastatin inhibited the invasion and metastasis of HOP-92 cells in the lungs of chick embryos, whereas no change was observed in the liver (Fig. 5b). A mouse xenograft model was used to analyze the anticancer effects of statins in mammals. HOP-92-Luc cells expressing the luciferase gene were transplanted into mice and in vivo imaging system (IVIS) images were obtained at one and two weeks (Fig. 6a). In the control group, bioluminescent signals derived from HOP-92-Luc were observed in all individuals one and two weeks after transplantation (Fig. 6b). In contrast, in the atorvastatin-treated group, no clear HOP-92-Luc signal was observed in three out of five mice one week after transplantation, and a signal was observed in only two mice two weeks after transplantation. A comparison of HOP-92-Luc-derived luminescence intensity showed that HOP-92 cells tended to have lower signal one week after transplantation in the atorvastatin group than in the control group, although the difference was not statistically significant (Fig. 6b).

## Discussion

Mesenchymal-like cells, characterized by high vimentin expression and the absence of membrane-bound E-cadherin, tend to be more sensitive to statins^21^. However, the mechanisms underlying the association between epithelial-mesenchymal phenotypes and statin sensitivity remain poorly understood. We therefore sought to elucidate this relationship by examining the association between statin sensitivity and YAP/TAZ activity in six NSCLC cell lines exhibiting varying epithelial-mesenchymal phenotypes. Our findings revealed that atorvastatin more effectively inhibits YAP/TAZ nuclear localization in mesenchymal-like NSCLC cells compared with that in epithelial-like cells. Moreover, siRNA-mediated co-silencing of YAP and TAZ significantly reduced proliferation across all six NSCLC cell lines, irrespective of their epithelial-mesenchymal phenotype. These results suggest that YAP/TAZ activity plays a critical role in NSCLC cell proliferation and that statins exert their anticancer effects, at least in part, by inhibiting YAP/TAZ signaling in mesenchymal-like NSCLC cells.

As we previously reported^27^, survival is decreased in mesenchymal-like cells when GGPP synthase is knocked down by siRNA, whereas epithelial-like cells are less affected. This observation suggests a differential reliance on GGPP for cell survival. GGPP is known to activate Rho GTPases, which in turn promote the nuclear localization of YAP/TAZ^17^. Therefore, mesenchymal-like cells may be more susceptible to GGPP depletion-induced disruption of YAP/TAZ nuclear localization. Statins promote the phosphorylation of YAP/TAZ, thereby inhibiting their nuclear localization and transcriptional activity^17,28^. Indeed, statins promoted YAP phosphorylation in mesenchymal-like HOP-92 cells but negligibly affected epithelial-like NCI-H322M cells (Supplementary Fig. S8). These findings indicate a differential cellular response to statin-induced YAP/TAZ phosphorylation, which may underlie variations in statin sensitivity between mesenchymal and epithelial NSCLC phenotypes. However, the molecular basis for this differential response remains unclear and warrants further investigation.

Although mesenchymal-like NSCLC cells generally exhibited greater sensitivity to statins than epithelial-like cells, we observed variability even among the mesenchymal-like cells. Notably, RERF-LC-MS cells were less responsive to atorvastatin compared with the other two mesenchymal-like cells, as treatment with 3 µM atorvastatin did not alter YAP/TAZ nuclear localization and produced only a modest reduction in cell viability. Furthermore, YAP/TAZ double knockdown induced only limited growth inhibition in RERF-LC-MS compared with that in the other mesenchymal-like cells, possibly reflecting a reduced dependency on YAP/TAZ signaling in this cell line. This lower reliance may partly account for the modest response to statin treatment, and suggests that YAP/TAZ-independent mechanisms may also contribute to the effects of statins.

We found that the factors contributing to cell growth inhibition upon siRNA-mediated knockdown of either YAP or TAZ varied across different cell lines. Although the exact reason for this variability remains unclear, it may depend on cell line-specific characteristics, experimental conditions, or differential activation levels of YAP versus TAZ. An analysis of nuclear YAP/TAZ expression in hepatocellular carcinoma tissues revealed that in 70% of patient-derived samples, either YAP or TAZ, but not both, was predominantly localized in the nucleus^29^. Additionally, in hepatocellular carcinoma cell lines treated with siRNA, growth inhibition was more pronounced with siYAP in some lines, and with siTAZ in others^29^. These observations suggest that the relative activity of YAP and TAZ varies between cancer types and among individual cell lines. Although statins inhibit both YAP and TAZ, the resulting anticancer effects may be more evident against the protein that is more active within a given cell line. Conversely, combined knockdown of both YAP and TAZ consistently suppressed growth in all NSCLC cell lines. Interestingly, YAP knockdown led to compensatory upregulation of TAZ in most cell lines, whereas TAZ knockdown did not affect YAP expression, indicating an asymmetric regulatory mechanism between YAP and TAZ. These findings underscore the potential importance of simultaneously targeting both YAP and TAZ in cancer therapy to overcome compensatory signaling and enhance therapeutic efficacy.

Both YAP/TAZ are frequently activated in various types of cancers, where they facilitate tumor progression and poor prognoses^16^. Furthermore, YAP expression in NSCLC confers drug resistance to EGFR tyrosine kinase inhibitors^30^. Both YAP and TAZ promote the transcription of genes involved in cell survival, proliferation, motility, and resistance to apoptosis. The YAP/TAZ target genes downregulated in statin-treated HOP-92 cells promote cancer progression. In particular, the extracellular matrix-associated proteins, CYR61 and CTGF, bind to various integrin receptors and activate downstream signaling pathways that promote cancer cell proliferation, motility, and EMT^31,32^. The receptor tyrosine kinase, AXL, also promotes EMT^33^ and it has been linked to tumor progression in lung adenocarcinoma^34^. In addition, the transcriptional coactivator, ANKRD1, activates NF-κB to promote metastasis^35^, and the glucose transporter, SLC2A1, which is overexpressed in lung cancer, contributes to tumor growth and survival by activating energy metabolism^36,37^. Therefore, the reduced expression of these genes by statin-mediated inhibition of YAP/TAZ might contribute to suppressed proliferation, motility, and metastasis of mesenchymal-like cells.

The EMT is promoted by YAP/TAZ^38,39^ so cells with high YAP/TAZ activity should have a mesenchymal-like phenotype. Indeed, the analysis of RNA expression data from 143 NSCLC cell lines revealed a trend toward higher YAP/TAZ target gene expression in mesenchymal-like cells (Supplementary Fig. S5). This is supported by the results of gene expression data downloaded from 181 lung cancer cell lines, which found enriched YAP/TAZ target genes in mesenchymal lung cancers^40^. Therefore, YAP/TAZ activation probably contributes to the malignant behavior of mesenchymal NSCLC cells, and YAP/TAZ inhibition by statins might be a useful anticancer strategy.

We assessed the anticancer effects of atorvastatin in mesenchymal NSCLC in vivo, using CAM assays of embryonated chicken eggs as metastatic models. Because chicken embryos are immunodeficient, cancer cells can be viable on the chorioallantoic membrane, and the process of cancer progression, including proliferation, invasion, angiogenesis, and metastasis, can be faithfully reproduced^26^. Statin inhibited HOP-92 cell metastasis to the lungs in chick embryos, but had no significant effects on the liver. Therefore, the ability of statins to inhibit metastasis might differ depending on target organs. However, further verification is needed to clarify this. We delivered HOP-92 cells to xenograft mouse models via intrapleural inoculation. The cells disappeared in both control and statin groups without significant differences, but the rate tended to be faster in the group administered with statin. However, this ectopic model did not mimic NSCLC growth in the lung parenchyma. Therefore, the anticancer effects of statins require verification in an orthotopic NSCLC model that more closely resembles the actual pathology of the disease.

Our results suggest that atorvastatin suppresses the proliferation, motility, invasion, and metastasis of mesenchymal-like NSCLC by inhibiting YAP/TAZ activity, which confer a metastatic phenotype and chemoresistance. Therefore, statins might be appropriate to treat highly malignant mesenchymal NSCLCs with high YAP/TAZ activity and might complement existing therapies.

## Methods

### Cell culture

We obtained HOP-92 and NCI-H322M cell lines derived from human NSCLCs (DCTD Tumor Repository, National Cancer Institute, Frederick, MD, USA) and A549 (JCRB0076), LU99 (JCRB0080), RERF-LC-MS (JCRB0081), and ABC-1 (JCRB0815) (Japanese Collection of Research Bioresources [JCRB], Osaka, Japan). All cells were cultured at 37 °C under a 5% CO_2_ atmosphere in RPMI 1640 medium (Thermo Fisher Scientific, Waltham, MA, USA) containing 10% fetal bovine serum (FBS; Biosera, Boussens, France) and 100 units/mL of penicillin G and 100 µg/mL of streptomycin (Fujifilm Wako Pure Chemical, Osaka, Japan).

### Cell proliferation assay

The cells grown in 96-well plates were treated with 0.3–30 µM atorvastatin (Sigma-Aldrich, St. Louis, MO, USA) diluted in dimethyl sulfoxide (DMSO; Fujifilm Wako Pure Chemical) for 48 h. Cells treated with equal volumes of DMSO (0.3%) acted as vehicle controls. Cell viability was determined using the cell counting kit-8 (CCK-8; Dojindo, Kumamoto, Japan) assay according to the manufacturer’s instructions.

### Western blotting

Cell lysate prepared using the CelLytic M solution (Sigma-Aldrich) was centrifuged at 16,000 × g for 15 min at 4 °C and the supernatant was collected. Protein concentrations were determined using the bicinchoninic acid protein assay kit (Thermo Fisher Scientific). Proteins were incubated with Laemmli sample buffer (Bio-Rad, Hercules, CA, USA) at 90 °C for 3 min. NuPAGE 4–12% Bis–Tris gel (Thermo Fisher Scientific) was used for electrophoresis, and 5 µg of protein lysates was loaded per lane. After electrophoresis, the proteins were transferred to iBlot gel transfer stack nitrocellulose (Thermo Fisher Scientific) using an iBlot gel transfer device (Thermo Fisher Scientific). The nitrocellulose membranes were blocked with 5% (w/v) skim milk (Morinaga Milk Industry, Tokyo, Japan) in Tris-buffered saline containing 0.05% Tween 20 (TBS-T). Anti-E-cadherin rabbit monoclonal antibody (1:1000 dilution, 24E10; Cell Signaling Technology, Beverly, MA, USA), anti-vimentin mouse monoclonal antibody (1:1000 dilution, 5G3F10; Cell Signaling Technology), anti-YAP/TAZ rabbit monoclonal antibody (1:1000 dilution, D24E4; Cell Signaling Technology), and anti-phospho-YAP rabbit polyclonal antibody (1:1000 dilution, #4911S; Cell Signaling Technology) were used as primary antibodies. Anti-glyceraldehyde-3-phosphate dehydrogenase (GAPDH) rabbit monoclonal antibody (1:1000 dilution, 14C10; Cell Signaling Technology) was used as the loading control. The membranes were washed with TBS-T and incubated with horseradish peroxidase-conjugated secondary antibodies, anti-mouse IgG goat antibody (R&D Systems, Minneapolis, MN, USA) or anti-rabbit IgG goat antibody (SeraCare, Milford, MA, USA) for 1 h. The membrane was washed and incubated with Clarity western ECL substrate chemiluminescence detection reagent (Bio-Rad) for 5 min. Proteins were detected using a C-DiGit blot scanner (Li-Cor Biosciences, Lincoln, NE, USA).

### Immunofluorescence microscopy

Cells grown on coverslips in 24-well plates were fixed using 2% paraformaldehyde (Nacalai Tesque, Kyoto, Japan) for 30 min, washed with phosphate buffered saline (PBS), and permeabilized using 0.1% Triton-X-100 (Nacalai Tesque) in PBS for 15 min. After washing with PBS, non-specific protein binding was blocked with 2% bovine serum albumin (Fujifilm Wako Pure Chemical) for 15 min. The cells were then incubated with the following primary antibodies for 1 h: anti-E-cadherin rabbit monoclonal antibody (1:200 dilution, 24E10; Cell Signaling Technology), anti-vimentin mouse monoclonal antibody (1:200 dilution, ab8978; Abcam, Cambridge, UK), and anti-YAP/TAZ rabbit monoclonal antibody (1:200 dilution, D24E4; Cell Signaling Technology). After washing with PBS, the cells were incubated with CF488A goat anti-rabbit IgG and CF568 goat anti-mouse IgG (1:200 dilution; Biotium, Hayward, CA, USA) for 15 min. Nuclei were stained with Hoechst 33342 (5 µg/mL; Nacalai Tesque) for 15 min. The cells were washed and transferred to slides in Fluoromount/Plus mounting medium (Diagnostic Biosystems, Pleasanton, CA, USA). Images were captured using a Fluoview FV10i confocal microscope (Olympus, Tokyo, Japan).

### Wound healing assay

Cells were grown to confluence in 96-well plates, and the surface was scratched with a sterile pipette tip. Cells were incubated in serum-free medium with 0.1–1 µM atorvastatin or DMSO for 48 h. Images of the wounded areas were captured and analyzed using the ImageJ software (National Institutes of Health, Bethesda, MD, USA). Wound closure (%) was calculated using the following formula: (Wound area 0 h−48 h)/Wound area 0 h × 100.

### Cell tracking analysis

Cells were seeded at low density in 96-well plates and treated with 0.1 µM atorvastatin or DMSO. Time-lapse images were captured every 2 h for 48 h using IncuCyte ZOOM (Essen BioScience, Ann Arbor, MI, USA) and analyzed using the ImageJ software (National Institutes of Health).

### Invasion assay

Matrigel (#356234; Corning, Corning, NY, USA) in serum-free medium was added to transwell inserts (#3464; Corning) in 24-well plates and incubated at 37 °C for 1 h. Cells (5 × 10^4^/well) suspended in serum-free medium were placed in transwell inserts and 10% FBS-supplemented medium was placed in the bottom well. Thereafter, atorvastatin (0.1, 0.3, and 1 µM) or DMSO (vehicle) was added to the cell suspension and incubated at 37 °C for 48 h. The cells inside the transwell inserts were gently removed and those on the lower surface of the inserts were fixed in 2% paraformaldehyde and stained with 0.1% crystal violet (Fujifilm Wako Pure Chemical). The crystal violet bound to the cells was eluted with methanol, and the absorbance at 595 nm was measured using a Sunrise-Basic microplate reader (Tecan Austria GmbH, Grödig, Austria) to quantify the invasive cells.

### Real-time quantitative polymerase chain reaction (qPCR)

Total cellular RNA was extracted using an ISOSPIN cell and tissue RNA kit (Nippon Gene, Tokyo, Japan). cDNA was synthesized from 1 µg total RNA using ReverTra Ace qPCR RT with a gDNA remover kit (Toyobo, Osaka, Japan). Real-time PCR was performed using a LightCycler rapid thermal cycler system (Roche Diagnostics, Basel, Switzerland) with LightCycler FastStart DNA MasterPLUS SYBR Green I mix (Roche Diagnostics), according to the manufacturer’s instructions. The primer sets used were designed using Primer3 software^41^ (Table 1). PCR conditions were as follows: 95 °C for 10 min; 45 cycles of 95 °C for 10 s, 60 °C for 10 s, and 72 °C for 15 s.

**Table 1:** Primer sequences for qRT-PCR.

Gene	Primer sequence	Product size (bp)
*ANKRD1*	5′-ACGCCAAAGACAGAGAAGGA-3′	154
	5′-TTCTGCCAGTGTAGCACCAG-3′	
*AXL*	5′-GACGGGTCTGTGTCCAATCT-3′	157
	5′-ACGAGAAGGCAGGAGTTGAA-3′	
*CTGF* (*CCN2*)	5′-GTTCCAAGACCTGTGGGATG-3′	165
	5′-TGGAGATTTTGGGAGTACGG-3′	
*CYR61* (*CCN1*)	5′-AGCTCAGTCAGAGGGCAGAC-3′	137
	5′-GTTCTTGGGGACACAGAGGA-3′	
*GAPDH*	5′-GAGTCAACGGATTTGGTCGT-3′	238
	5′-TTGATTTTGGAGGGATCTCG-3′	
*SLC2A1*	5′-GGGCCAAGAGTGTGCTAAAG-3′	121
	5′-AACAGCTCCAGGATGGTGAC-3′	

### YAP/TAZ siRNA transfection

Predesigned siRNAs targeting YAP (siYAP: siRNA ID#s20368, siYAP#2: #s20366), TAZ (siTAZ: #s24789, siTAZ#2: #s24788) and scrambled control siRNA (#4390843) were obtained from Thermo Fisher Scientific. In 96-well plates, cells (3 × 10^3^ cells/well, exception A549 with 1 × 10^3^ cells/well) were reverse-transfected with 10 nM siRNAs using Lipofectamine RNAiMAX reagent (Thermo Fisher Scientific) according to the manufacturer’s instructions. Under siYAP + siTAZ conditions, YAP siRNAs and TAZ siRNAs (5 nM each) were added, then cell proliferation was assessed at 24, 72, and 120 h after transfection using CCK-8 assays (Dojindo) according to the manufacturer’s instructions. Seventy-two hours after siRNA transfection, half of the medium was replaced with medium containing siRNA and transfection efficiency was assessed using western blotting with an anti-YAP/TAZ rabbit monoclonal antibody (1:1000 dilution; D24E4; Cell Signaling Technology).

### CAM assays

Fertilized chicken eggs were developed in an incubator (Autoelex, Ginhae City, South Korea) at 37.5 °C with 60% relative humidity. HOP-92 cells (1 × 10^6^/egg) were suspended in a mixture of 25 µL RPMI complete medium and 25 µL Matrigel and inoculated on the CAM of 8-day-old chicken eggs. Atorvastatin (1 µM) was added to the cell suspension in the statin-treated group, while DMSO was added in the control group. The eggs were returned to the incubator at the end of the experiment. One hundred microliters of atorvastatin or DMSO diluted in RPMI 1640 medium was topically administered to the CAM of 11-day-old and 14-day-old chicken eggs. Seventeen-day-old embryos were euthanized by decapitation, and the lungs and liver were harvested. DNA was extracted from the lung and liver tissues of chicken embryos using ISOSPIN tissue DNA kit (Nippon Gene), and the amounts of human Alu and chicken *GAPDH* were quantified using real-time PCR. The primer sequences were as follows: human Alu (sense 5′-ACG CCT GTA ATC CCA GCA CTT-3′ and antisense 5′-TCG CCC AGG CTG GAG TGC A-3′), chicken GAPDH (sense 5′-GAG GAA AGG TCG CCT GGT GGA TCG-3′ and antisense 5′-GGT GAG GAC AAG CAG TGA GGA ACG-3′)^42^.

### Generation of a luciferase-expressing HOP-92 cell line

Red firefly luciferase cDNA was amplified using the pMCS-Red Firefly Luc vector (Thermo Fisher Scientific). The amplified red firefly luciferase cDNA was subcloned into the CSII-CMV-MCS-IRES2-Bsd vector (RIKEN BioResource Center, Tsukuba, Japan) to construct CSII-CMV-RFLuc-Bsd. CSII-CMV-RFLuc-Bsd plasmids were co-transfected into HEK293T cells with pCAG-HIVgp and pCMV-VSV-G-RSV-Rev (RIKEN BioResource Center) for lentivirus production. After 48 h of incubation, the medium containing lentivirus was collected. The HOP-92-Luc cell line was obtained after infection with the lentivirus, and selection was performed in a medium containing 10 µg/mL blasticidin.

### Tumor mouse model

The animal experimental protocol was approved by the Animal Experiment Committee of the Aichi Medical University (#2023-6). All experiments were performed in accordance with the ARRIVE guidelines and relevant institutional and national regulations. All invasive procedures were performed under 2% isoflurane inhalation anesthesia. Eight-week-old male BALB/c Slc-nu/nu mice (Japan SLC, Shizuoka, Japan) were maintained under specific pathogen-free conditions. The mice were divided into control and statin treatment groups. Mice in the statin-treated group were subcutaneously implanted with osmotic pumps (ALZET, Cupertino, CA, USA) to systemically release atorvastatin (10 mg/kg/day) dissolved in DMSO. The mice in the control group were implanted with osmotic pumps containing DMSO. Two weeks after pump implantation, all mice were inoculated with HOP-92-Luc cells (1 × 10^6^/body) in the pleural cavity. After one and two weeks, 150 mg/kg D-luciferin (Promega, Madison, WI, USA) was injected into the pleural cavity of the mice. Fifteen minutes after injection, tumor survival was monitored using IVIS (PerkinElmer, Waltham, MA, USA). The signals at the tumor site were quantified as photons per second using the Living Imaging software (PerkinElmer). Euthanasia was performed by cervical dislocation under isoflurane anesthesia 14 days after cell inoculation.

### Statistical analyses

Statistical analyses were performed using the SPSS v.28 software (IBM Corp., Armonk, NY, USA). Data were compared using two-tailed Welch’s t-test or Dunnett’s test for multiple comparisons. *p*-values < 0.05 were considered statistically significant.

## Supplementary Information

Supplementary Information.

## Data Availability

All data are available in the manuscript. Further data will be provided by T. I. upon request.
