# Supplementary material for: Atorvastatin exhibits anticancer effects by inhibiting YAP/TAZ activity in mesenchymal-like non-small cell lung cancer
Source: Sci Rep. 2025 Aug 18;15:30167. doi: 10.1038/s41598-025-15624-2 (PMC12361482; doi:10.1038/s41598-025-15624-2)
Supplement: Supplementary file 1 — Supplementary Information. [file 41598_2025_15624_MOESM1_ESM.pdf]

Supplementary Figure 1

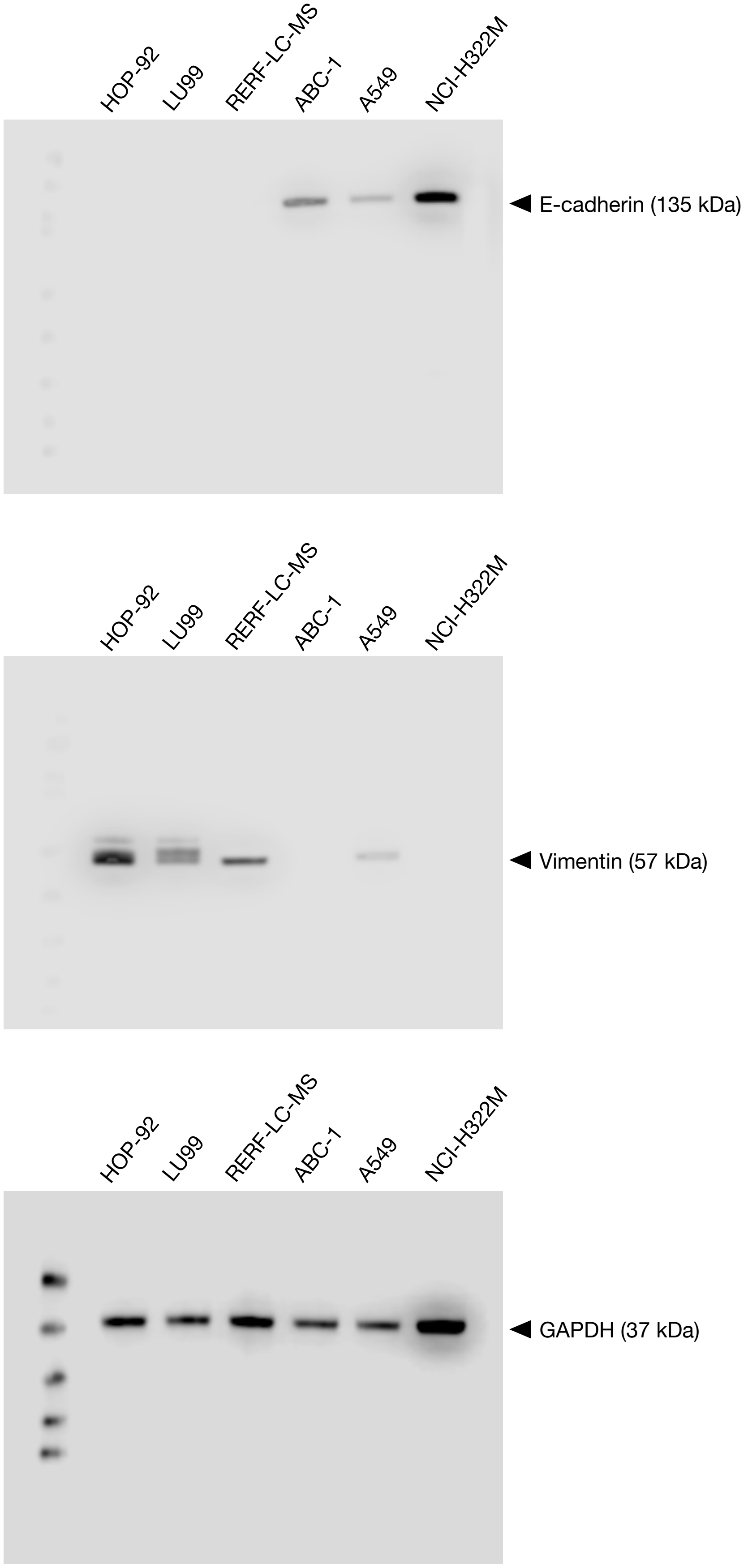

Supplementary Fig. S1. E-cadherin and vimentin levels in NSCLC cell lines. E-cadherin and vimentin levels in cell lines were determined using western blotting. GAPDH was used as the loading control. Original western blot images are shown.

Supplementary Figure 2

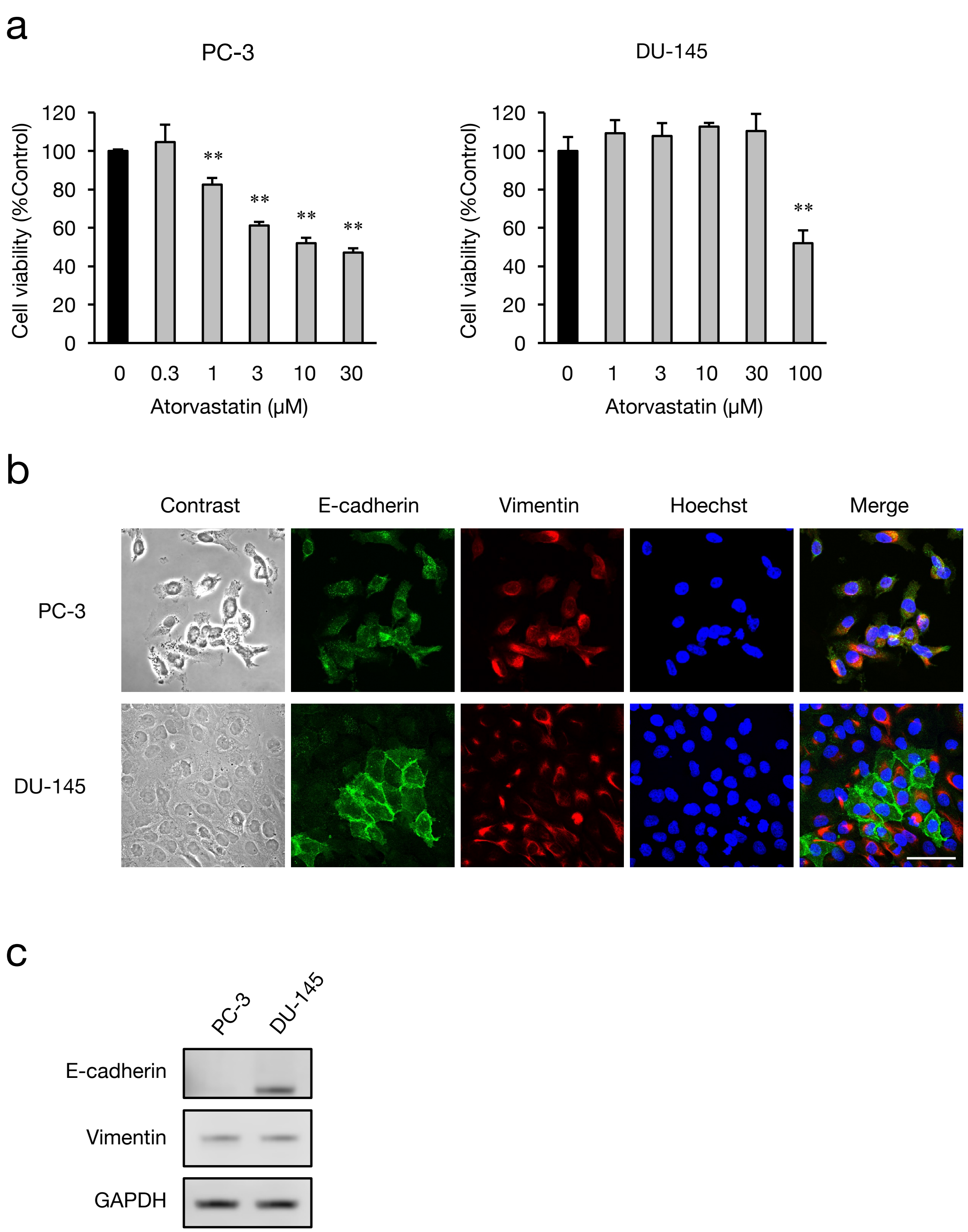

Supplementary Fig. S2. Sensitivity to atorvastatin and expression of E-cadherin and vimentin in prostate cancer cell lines. (**a**) Prostate cancer cell lines, PC-3 and DU-145, were treated with 0.3–100 μM atorvastatin or DMSO vehicle control, and cell viability was determined at 48 h using a CCK-8 assay. Each value represents the mean ± SD ($n = 3$). Data were analyzed using Dunnett's test: $^{**}p < 0.01$ compared to the vehicle control group. (**b**) Images of cells immunostained for E-cadherin (green), vimentin (red), and Hoechst 33342 (blue). Scale bar: 50 μm. (**c**) E-cadherin and vimentin levels in the cell lines were determined using western blotting. GAPDH was used as a loading control. Cropped images of the western blot are shown and original uncropped blots are presented in Supplementary Fig. S3.

Supplementary Figure 3

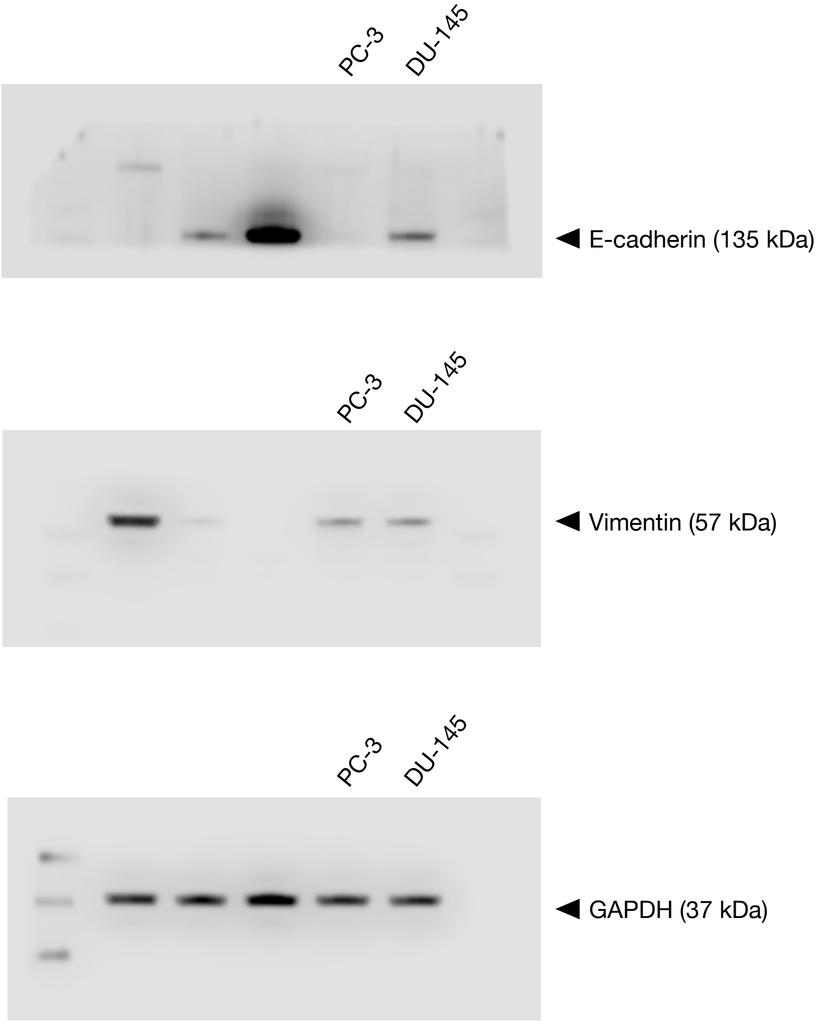

Supplementary Fig. S3. E-cadherin and vimentin levels in prostate cancer cell lines. E-cadherin and vimentin levels in cell lines were determined using western blotting. GAPDH was used as the loading control. Original western blot images are shown.

Supplementary Figure 4

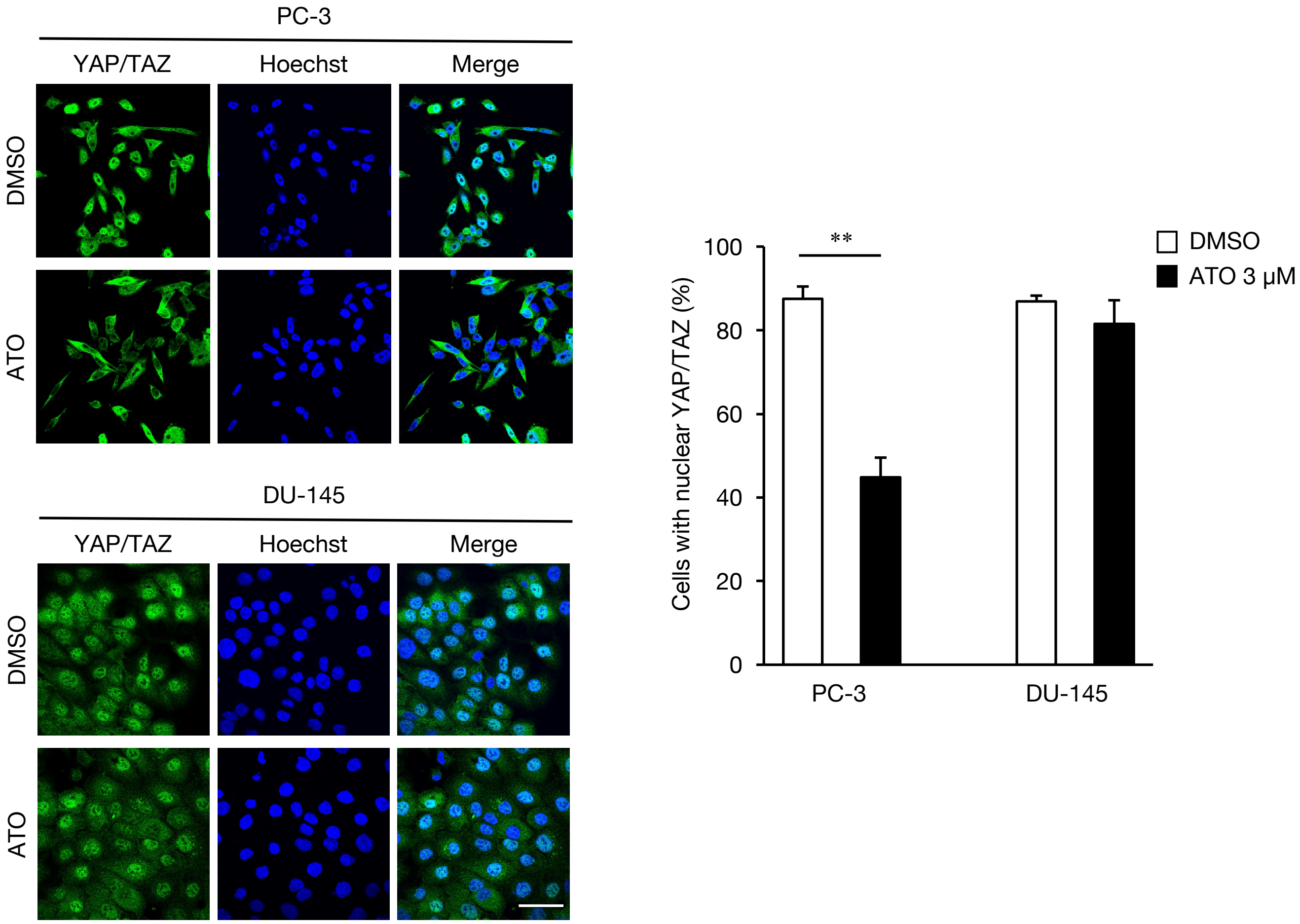

Supplementary Fig. S4. Effect of atorvastatin on YAP/TAZ activity in prostate cancer cell lines. Representative images of cells immunostained for YAP/TAZ (green) and Hoechst 33342 (blue) in PC-3 and DU-145 cells treated with 3 μM atorvastatin (ATO) or DMSO for 24 h. Bars show quantification of cells with nuclear YAP/TAZ. Each value represents the mean ± SD (n = 3; 300 cells were scored). Data were analyzed using two-tailed Welch's t-test: $^{**}p < 0.01$ compared to the vehicle control group. Scale bar: 50 μm.

Supplementary Figure 5

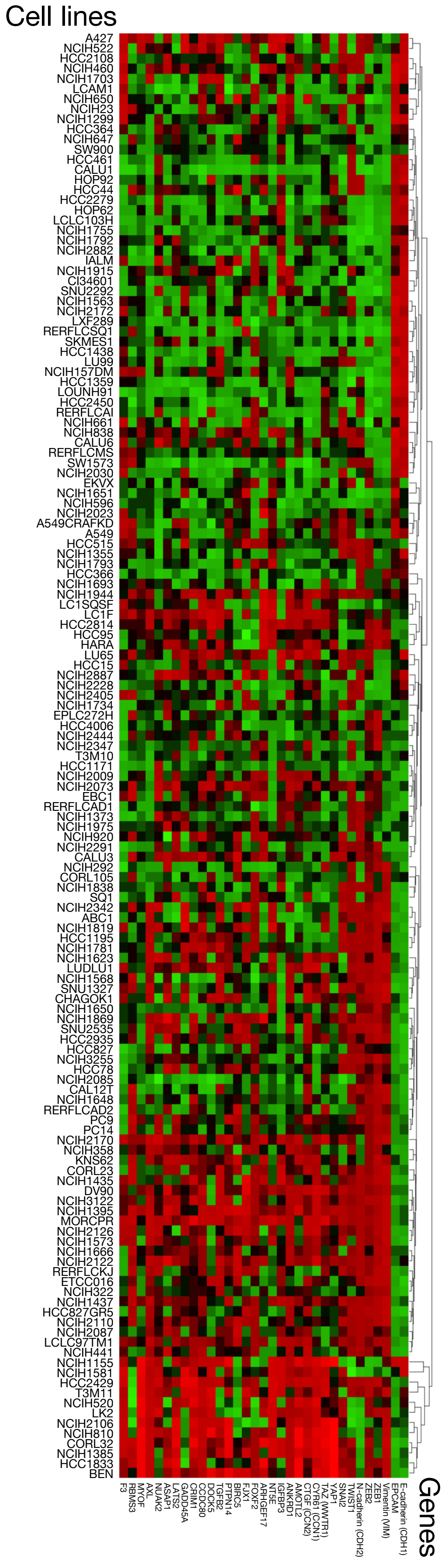

Supplementary Fig. S5. Correlation of YAP/TAZ target gene expression with mesenchymal cell markers in NSCLC cell lines. Batch-corrected RNA expression data of epithelial markers (*CDH1* and *EPCAM*), mesenchymal markers (*VIM*, *ZEB1*, *ZEB2*, *CDH2*, *TWIST1*, *SNAI2*), *YAP1*/*WWTR1*, and YAP/TAZ target genes were downloaded from the Cancer Cell Line Encyclopedia (CCLE) database (https://depmap.org/portal) for cell lines with lineage subtypes of NSCLC (n = 143). Cell lines were clustered based on the RNA expression levels. Heatmaps showing Z scores of gene expression were generated using Heatmapper (http://www.heatmapper.ca/expression/). Z scores calculated based on gene expression levels are shown in green (high) and red (low). We applied clustering and distance measurements using average linkage and Pearson methods, respectively.

Supplementary Figure 6

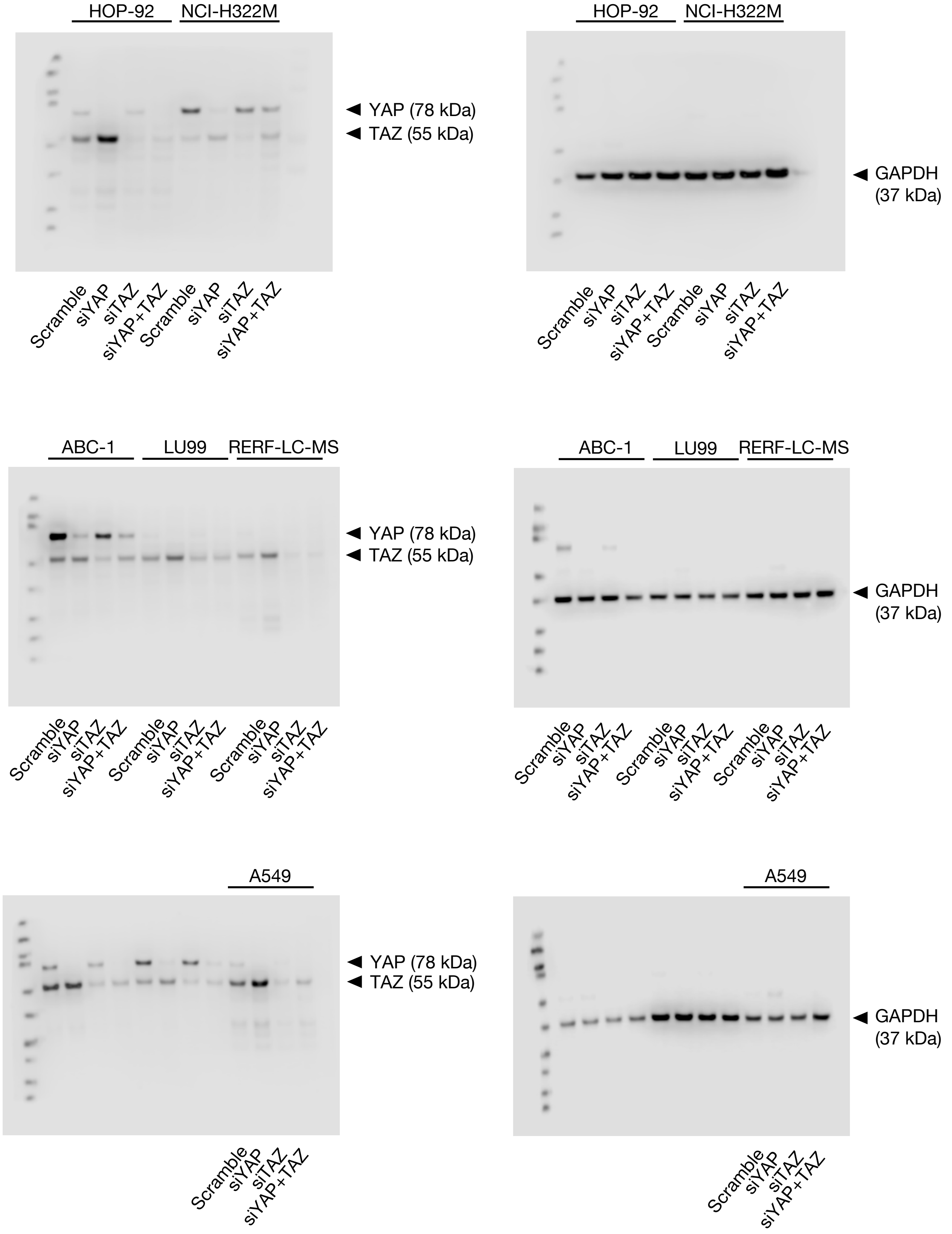

Supplementary Fig. S6. YAP and TAZ protein expression in NSCLC cell lines. YAP and TAZ levels in cells treated with siRNA targeting YAP and/or TAZ or scrambled control were determined using western blotting. GAPDH was used as the loading control. Original western blot images are shown.

Supplementary Figure 7

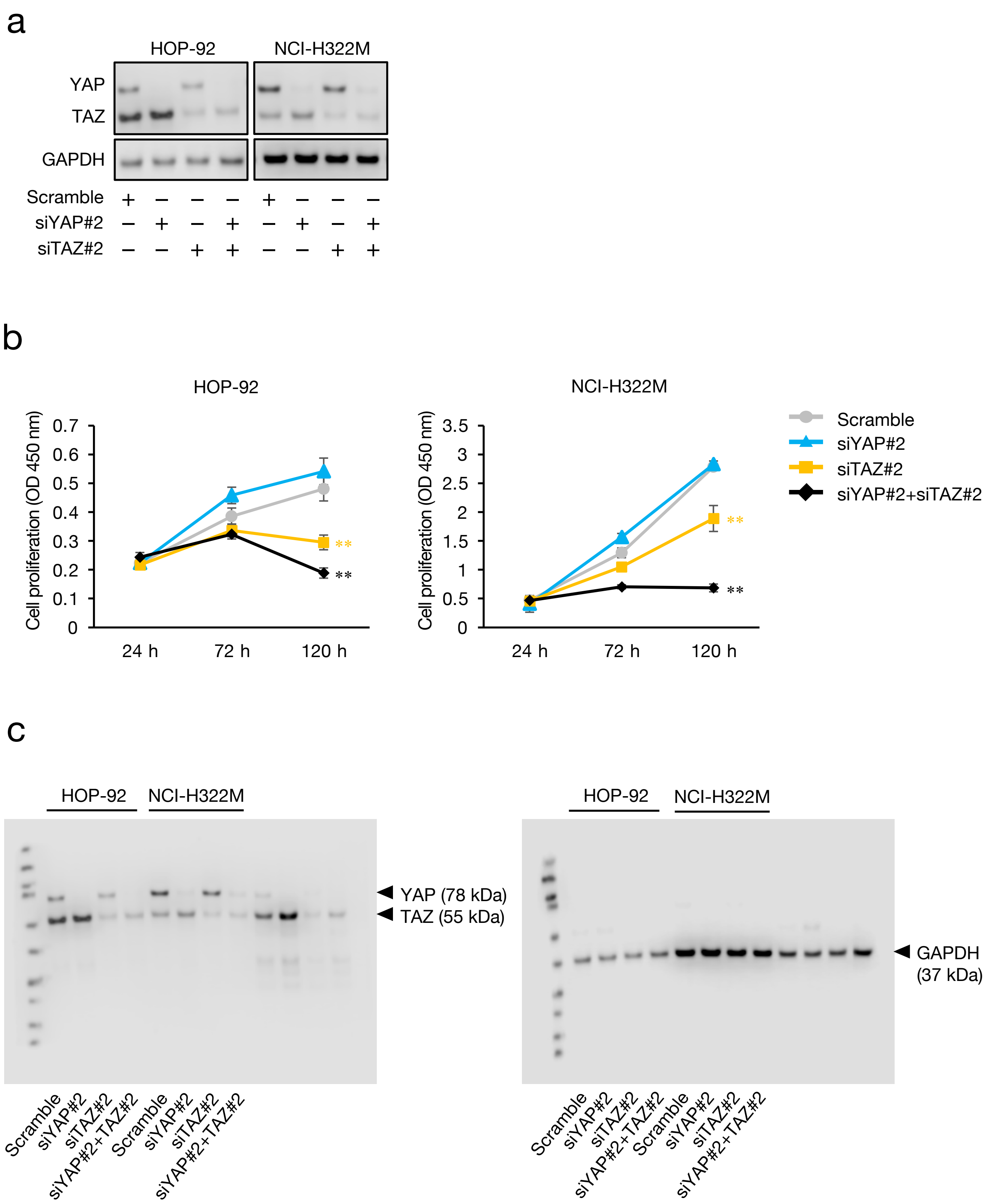

Supplementary Fig. S7. Effect of YAP/TAZ knockdown in NSCLC cell lines by different siRNA. (**a**) YAP and TAZ protein expression in HOP-92 and NCI-H322M cells treated with siRNA targeting YAP and/or TAZ or scrambled control for 72 h. GAPDH was used as a loading control. Cropped images of the western blot are shown and original uncropped blots are presented in Supplementary Fig. S7c. (**b**) Growth curve of HOP-92 and NCI-H322M cells treated with siRNA targeting YAP and/or TAZ or scrambled control. Each value represents the mean ± SD (n = 3). Data were analyzed using Dunnett's test: ***p* < 0.01, compared to the scrambled control group. (**c**) Original blot images are shown.

Supplementary Figure 8

a

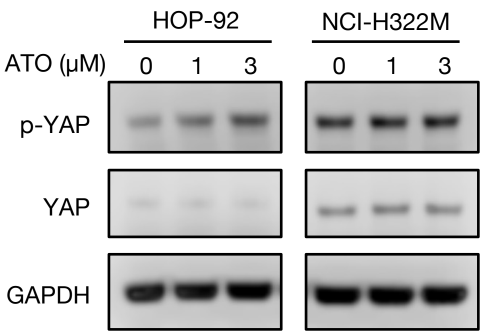

b

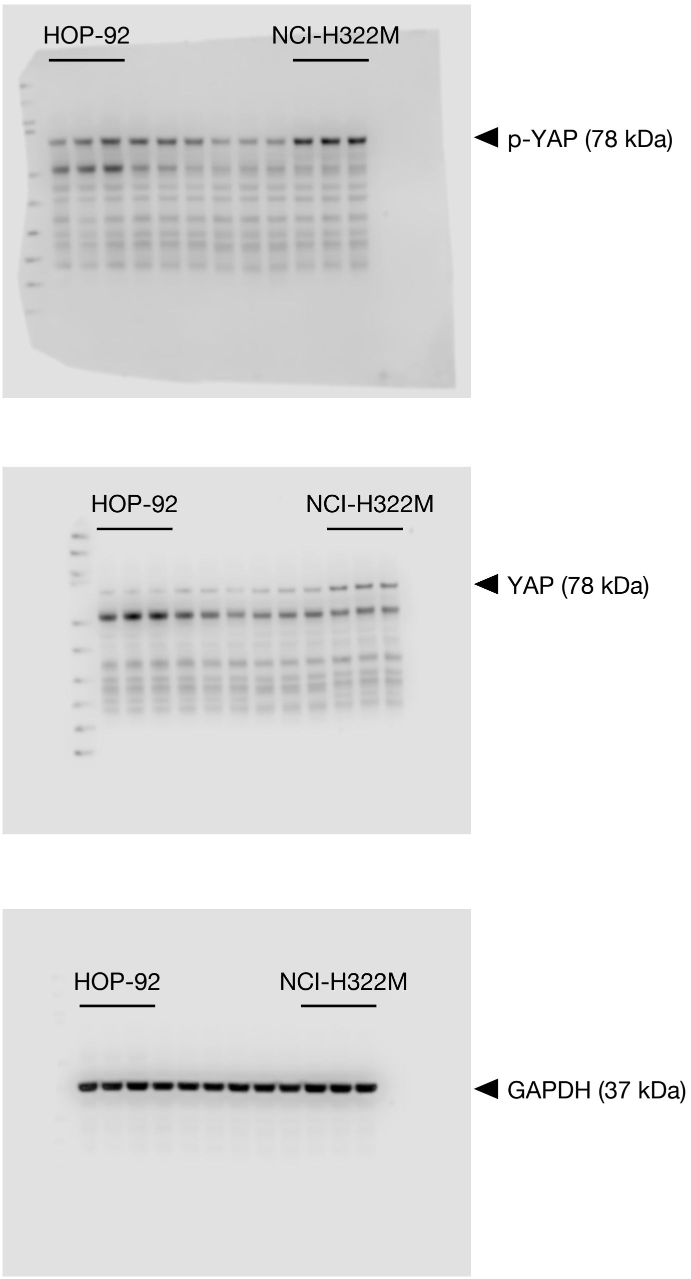

Supplementary Fig. S8. Effect of atorvastatin on YAP phosphorylation in NSCLC cell lines. (**a**) Cropped images of the western blot of YAP and its phosphorylation status in HOP-92 and NCI-H322M cells treated with 1 µM and 3 µM atorvastatin (ATO) or DMSO for 24 h. GAPDH was used as the loading control. (**b**) Original blot images are shown.
